# Influence of High-Frequency Repetitive Transcranial Magnetic Stimulation on Neurobehavioral and Electrophysiology in Patients with Disorders of Consciousness

**DOI:** 10.1155/2022/7195699

**Published:** 2022-11-16

**Authors:** Jian-Min Chen, Qing-Fa Chen, Zhi-Yong Wang, Yang-Jia Chen, Nan-Nan Zhang, Jian-Wen Xu, Jun Ni

**Affiliations:** ^1^Department of Rehabilitation Medicine, The First Affiliated Hospital of Fujian Medical University, Fujian, China; ^2^Department of Rehabilitation, Fujian Medical University Union Hospital, Fujian, China; ^3^Department of Rehabilitation Medicine, The First Affiliated Hospital of Guangxi Medical University, Guangxi, China

## Abstract

**Objective:**

High-frequency repetitive transcranial magnetic stimulation (HF-rTMS) has been proposed as a promising therapeutic intervention for patients with disorders of consciousness (DOC). However, its therapeutic effects in the literature are inconsistently documented. The primary aim of this study was to explore the alterations in neural connectivity and neurobehavioral reactivity during rTMS modulation in patients with DOC. In addition, safety was investigated as a secondary aim.

**Methods:**

The presence of bilateral N20 components in DOC patients was determined by somatosensory-evoked potential (SEP) before enrollment in the study. A total of 64 patients were enrolled and randomly placed into the active and sham groups. Ultimately, 50 patients completed the study. Twenty-five patients in the active group underwent real HF-rTMS, and 25 patients in the sham group underwent sham HF-rTMS, which was delivered over the left dorsolateral prefrontal cortex (DLPFC). The outcome measures of performed pre- and postintervention included the latencies of the N20 and N20-P25 amplitudes of SEP, brainstem auditory-evoked potential (BAEP) grade, JFK Coma Recovery Scale-Revised (CRS-R) score, and Glasgow Coma Scale (GCS) score; any adverse events were recorded at any time during the intervention.

**Result:**

Following six weeks of treatment, a significant increase was observed in the total CRS-R and GCS scores, and the N20-P25 amplitudes of patients in the two groups were compared with that obtained from preintervention (all *p* values < 0.05). The waves of BAEP in the two groups also showed a trend toward normalized activity compared with preintervention grades (*p* values < 0.05). A significant decrease in the latencies of N20 (*p* values < 0.001) was observed in the active group compared with measurements obtained from preintervention, whereas no significant decrease was observed in the sham group (*p* values = 0.013). The improvement in total CRS-R scores (*p* values = 0.002), total GCS scores (*p* values = 0.023), and N20-P25 amplitudes (*p* values = 0.011) as well as the decrease in latencies of N20 (*p* values = 0.018) and change in BAEP grades (*p* values = 0.013) were significantly different between the two groups. The parameters in neural connectivity (N20-P25 amplitudes, N20 latencies, and BAEP grades) were significantly correlated with the total CRS-R and GCS scores at postintervention, and the changes of CRS-R before and after interventions have a positive relationship with N20-P25 amplitudes. No adverse events related to the rTMS protocol were recorded.

**Conclusion:**

Neural connectivity levels are affected by HF-rTMS and are significantly related to clinical responses in DOC patients with the presence of bilateral N20. The elevation of neural connectivity levels may lay a foundation for successful HF-rTMS treatment for DOC patients.

## 1. Introduction

More and more patients with disorders of consciousness (DOC) are surviving from brain injury due to the ongoing improvements in intensive care and emergency medicine [1]. As soon as these patients are medically stabilized, the attention of clinicians and families rapidly turns to planning for the needs to the recovery of consciousness [2, 3]. DOC is a highly challenging condition, and although multiple efforts have been made to facilitate recovery [4, 5], only rare treatment schemes have been recommended by authoritative institutions [6]. Currently, effective clinical protocols for managing patients with DOC are still lacking [3].

In recent years, significant attention has been paid to repetitive transcranial magnetic stimulation (rTMS), a noninvasive and painless technique that has produced many inspiring beneficial results in the research of neurological diseases, such as stroke, Parkinson's disease, and Alzheimer's disease [7, 8]. Some published studies have also shown the potential therapeutic effects of rTMS in therapeutic interventions for DOC [9, 10]. Jang and Kwon [11] reported that rTMS induced cognitive and neurophysiological modifications in one patient in a persistent vegetative state. Ge et al. [10] showed that 10 Hz rTMS of the dorsolateral prefrontal cortex could improve the state of awareness of DOC patients in a nonrandomized controlled trial. However, other studies have failed to provide evidence of any obvious therapeutic effects of such treatment when compared with the control groups. Cincotta et al. [12] assessed the effects of rTMS in 11 patients with DOC in a randomized sham-controlled study with a crossover design. In their study, significant differences were not observed in the JFK Coma Recovery Scale-Revised (CRS-R) and Clinical Global Impression Improvement (CGI-I) scale scores between the real or sham stimulation conditions. Naro et al. [13] examined the feasibility of a single session of 10 Hz rTMS over the DLPFC in patients with DOC and did not find any clinical improvement or neural connectivity changes at the group level [13].

On the other hand, there is still no consensus as to how best to measure the degree of consciousness impairment in noncommunicating patients [14] and assess the modulation effects of the interventions on DOC [15, 16]. These results have significant ethical and practical implications for the caregivers and clinicians of DOC patients regarding outcome prognostication, medical care, rehabilitation services, and resource allocation [17, 18]. The current gold standard for assessing consciousness states used in previous studies is based on standardized clinical rating scales that are critically reliant on behavior observation [11, 19]. The results of such assessments are often confounded by underlying sensorimotor impairment and unrecognized cognitive and language deficits. Perhaps more important is the fact that patients' behavioral abilities may fluctuate across time, thus causing misdiagnosis [20].

It is well known that consciousness is regulated by the activation of neural pathways. Connectivity is an important feature of neural pathways [20], and the disruption of pathway connectivity is related to the degree of consciousness breakdown [13], with a significant relationship to prognosis [21]. Recent findings have suggested that the response to rTMS in DOC patients is mediated by the neural networks preserved after insult [22]. With recent advances in computer instrumentation and signal processing over the past several years, the introduction of evoked potential (Ep) technologies has enabled the evaluation of the integrity of neural functional connectivity in a live human brain. EPs show increasing promise as powerful tools for assessing the severity of impairment and predicting the prognosis in patients with DOC [5], which are associated with a series of sensory events induced by the presence of specific sensory stimuli without being confounded by sedating medications and sleep [14]. This process can be used to avoid misjudgments caused by sensorimotor, verbal, and cognitive deficits [2]. More importantly, lengthy clinical practice has demonstrated that EPs can provide a reliable assessment of the connectivity of neural pathways [17]. In particular, the N20 and P25 responses to median nerve stimulation by somatosensory-evoked potential (SEP) have been shown in many studies to be the predictor of the responsiveness prognosis in DOC patients [23–26]. In other words, patients with a bilateral presence of the wave N20 and P25 responses to median nerve stimulation by SEP may be more likely to benefit from the treatment [27].

Based on the principle of neural plasticity, rTMS can strengthen the connectivity of neural pathways through a long-term potentiation-like mechanism [5, 28], stimulating arousability and functional integration within neural networks to facilitate the emergence of consciousness [29]. Although numerous previous studies have suggested the potential role of neural pathways in behavioral modifications caused by HF-rTMS, whether HF-rTMS influences neural connectivity levels has not yet been directly investigated. Herein, we propose a new method for selecting patients according to their SEP before study enrollment, presenting results from a sham-controlled trial examining whether rTMS over the DLPFC affects neural connectivity levels while improving the level of consciousness in patients experiencing DOC.

## 2. Methods

### 2.1. Participants

In our study, we included 50 patients with DOC who were consecutively admitted to the Department of Rehabilitation Medicine of The First Affiliated Hospital of Fujian Medical University from February 2020 to January 2022. The study was approved by the Ethics Committee of The First Affiliated Hospital of Fujian Medical University (approval number [2020]031). The entire study design and all procedures were performed in accordance with the Declaration of Helsinki. Written informed consent to participate in the study was obtained from the legal guardian of each patient, as patients were not deemed capable of giving consent. The http://chictr.org identifier is ChiCTR2000030419 (http://www.chictr.org.cn/showproj.aspx?proj=50162).

All patients enrolled in this study were 18–75 years of age, with an onset duration of 1–3 months, and met the diagnostic criteria for the vegetative state (VS) or minimally conscious state (MCS) when assessed with the CRS-R scale widely used to define levels of consciousness and monitor neurobehavioral recovery in patients [29]. Brain lesions were confirmed, and communicating hydrocephalus was ruled out by magnetic resonance imaging or computerized tomography scans. The exclusion criteria were the unilateral or bilateral absence of N20; unstable vital signs; epileptic history or EEG epileptiform activity; implanted pacemakers and severe dysfunction of heart, liver, or kidney; previous neurological or psychiatric disorders; acute pneumonia and other extreme complications; craniotomy or metallic implantation on the right side of the head; and any other safety contraindications to TMS.

**Figure 1:**
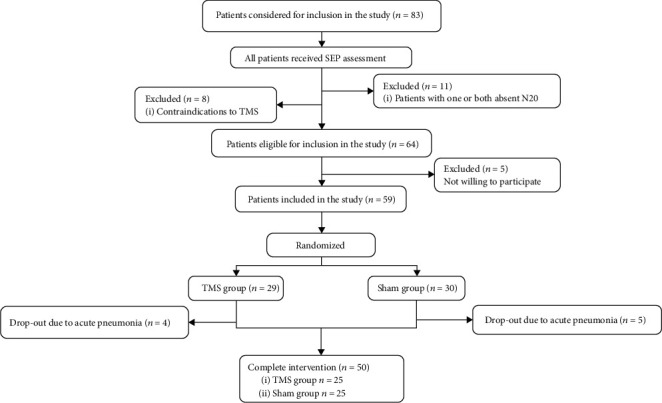
Flow chart of the study.

The first SEP was administered to patients before being enrolled in the study to ensure the bilateral presence of the N20 and P25 components. Participants were randomly divided into an active group and a sham group using a random number table. All participants received a similar routine medication (amantadine, antiepileptic, anti-inflammatory, etc.) and a rehabilitation course (hyperbaric oxygen, passive exercises, electrical nerve stimulation, etc.) during the trial. On this basis, participants in the active group were treated with real rTMS, whereas those in the sham group were treated with sham stimulation (Figure 1).

### 2.2. Stimulation Protocol

The rTMS was administered over five consecutive working days (from Monday to Friday) for six weeks. Stimulation intensity varied across this experiment was determined relative to the resting motor threshold (RMT) by stimulation of the M1 region corresponding to the right first dorsal interosseous (FDI) muscle representation (approximately position C3 of the 10/20 international electroencephalography system) and was recorded with an electromyogram amplifier module and surface electrodes. The patients were seated in a comfortable reclining chair and fitted with earplugs. The figure-8-shaped coil was placed at a tangent to the scalp, with the handle pointing backward and laterally at a 45° angle away from the midline. According to the International Federation of Clinical Neurophysiology Committee recommendations [30], the RMT intensity was defined as the minimum stimulus intensity that induces MEP greater than 50 *μ*V in at least five of 10 consecutive trials during muscle relaxation. The earplugs were inserted into the ears of patients, which continuously played a masking noise to prevent the interference of auditory potentials with TMS discharge during RMT measurement [12, 19, 28, 31, 32].

**Figure 2:**
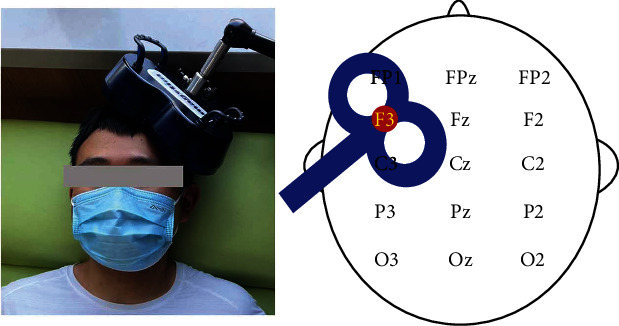
Site of stimulation: left dorsolateral prefrontal cortex.

The rTMS procedure consisted of a session of 1,000 pulses delivered in 10 trains of 10 Hz at an intensity of 90% RMT. Each train lasted 10 s with an intertrain pause of 60 s between each one. The coil was placed tangentially toward the scalp over the left DLPFC (position F3 of the 10/20 international electroencephalography system) for active stimulation. The junction region of the coil pointed backward and laterally at a 45°angle away from the midline [9, 13, 32, 33]. The placement of the coil is shown in Figure 2. The sham rTMS was delivered using the same protocol except that the coil was held at an angle of 90° to the scalp [34]. The protocol of stimulation was administered according to safety guidelines [35]. The rTMS was performed by a physical therapist who was blinded to the assessments (NNZ). A registered nurse or physician was required to be present at every rTMS session, ensuring that if a seizure occurred during or after rTMS, the patient would be treated in time [36]. The EEGs recorded biweekly were compared with the baseline EEG to identify possible patterns indicating an impending seizure. Structural MRIs were also completed postintervention; the MRIs were monitored by a neuroradiologist for changes from baseline, including hemorrhage and edema/toxic tissue.

### 2.3. Outcome Measures

The assessments were performed pre- and postintervention in a quiet room, with patients lying on a comfortable bed. The complete clinical examinations were performed by a trained clinician (ZYW). The electrophysiological parameters were recorded using a NeMus 2 evoked potential system (EB Neuro S.p.A., Florence, Italy) by rehabilitation physicians (YJC and JMC). All the assessments were blinded to this experimental design.

#### 2.3.1. Clinical Assessments

The clinical assessments in this study included the CRS-R and Glasgow Coma Scale (GCS). CRS-R is a standardized tool consisting of 23 organized items divided into six subscales addressing auditory, visual, motor, verbal, communication, and arousal processes. The subscales are comprised of hierarchically arranged items associated with the brainstem, subcortical, and cortical processes. The score in each CRS-R subscale is determined according to the presence or absence of specific responses to a sensory stimulus, with a higher total score indicating a greater level of consciousness [12, 37, 38]. GCS is a behavioral measure universally accepted as a gold standard for assessing the severity of a brain injury [2, 37, 39] and level of consciousness [40] [34] in terms of a patient's ability to respond to stimuli; eye opening (maximum 4 points), best motor response (maximum 6 points), and verbal response (maximum 4 points) are all measured. Each level of response is assigned a number and added together to provide a total score between 3 and 15 [41]; the worse the response, the lower the number [36]. Individual patients are best described by the three components of the Glasgow Coma Scale, whereas the derived total coma score can be used to characterize groups [17, 25, 39, 42].

#### 2.3.2. Electrophysiological Evaluations

The SEP was recorded through Ag-AgCl surface electrodes that were placed over the bilateral supraclavicular fossae (Erb's point), spinous process of the sixth cervical vertebrae (Cv6), frontal pole (Fpz), and each somatosensory cortex contralateral to stimulation (C3′, C4′) according to the International 10–20 system. The SSEPs were recorded after median nerve stimulation of the wrist (duration: 0.2 ms; stimulus rate: 4.0 Hz). The impedance was kept below 3 k*Ω*, and SSEP was amplified with a bandpass of 20–1000 Hz. At least 300 responses were averaged into each waveform and obtained three times (a total of 900 responses). Next, the absolute latencies of N20 and the amplitudes of N20-P25 peak-peak (N20-P25 amplitudes) were measured. N20 was defined as the major negative peak with a latency of about 20 ms after stimulations, and P25 was defined as the major positive peak following the N20 [26]. If the parameters were asymmetrical, the parameters on the more impaired side were recorded and used for analysis [27, 43].

Acoustic stimuli for BAEP were delivered through earphones. A masking white noise of 40 dB intensity was used on the contralateral side. Clicks of 100 us and 90–110 dB intensity was used at a rate of 10.7 Hz. At least three runs of 1,500 stimuli were averaged, and reproducibility was assessed by superimposing the traces. Recording electrodes were placed on bilateral mastoids (A1, A2), and the reference electrode was placed at the Fpz [18, 44]. The identification of waves for BAEP grading utilized Hall's classification as follows [25]: grade 1, normal; grade 2, mild abnormality, moderate waveform differentiation with the following possible problems: prolonged I, III, or (and) V wave peak latency, prolonged interpeak latency of the I-III, III-V, or (and) I-V waves, peak-to-peak latency ratio of III − V/I − III > 1, and V/I wave amplitude ratio < 0.15; grade 3, moderate abnormality, poor waveform differentiation, and poor repeatability with the following possible problems: prolonged peak latency of III or V waves and the disappearance of V waves; and grade 4, severe abnormality, presence of I waves only, or disappearance of all waveforms.

### 2.4. Statistical Analysis

Qualitative data were presented as numbers. The distribution of quantitative data was tested for normality using the Shapiro–Wilk test and for homogeneity of variances using Levene's test. Normally distributed variables were presented as the mean (standard deviation) and nonnormally distributed variables as the median (interquartile range). The Mann–Whitney *U* test, the independent samples *t*-test, and the Chi-squared test were used for comparisons of data between the two groups with the baseline. Intragroup differences in pre- and postintervention were tested using a two-tailed unpaired Student's *t*-test and paired Wilcoxon rank-sum test. The Spearman rank correlation was used to test for a significant association between the total GCS and CRS-R scores, total scale scores, and electrophysiological parameters. The effects of the experimental intervention (changes) were calculated by subtracting the baseline data from the data obtained from postintervention (6 weeks) between the groups and were compared using the Mann–Whitney *U* test and the independent samples *t*-test. A *p* value of 0.05 or less was considered statistically significant. All statistics were performed using the SPSS software (version 23.0, IBM Corporation, Armonk, NY, USA).

## 3. Results

Table 1 summarizes the demographic and clinical characteristics of all patients. Both groups were homogeneous for age, time since injury, etiology, total CRS-R, and GCS scale scores. Latencies of N20, N20-P25 amplitudes, and BAEP grade were also homogeneous at baseline for the active and sham groups (all *p* values > 0.05). All patients tolerated the study without complications, and no adverse effects were reported. The sample plots of SEP and BAEP in the two groups before and after interventions were provided in the Supplementary Materials (available here).

### 3.1. The Effects of rTMS on Clinical Assessment

**Figure 3:**
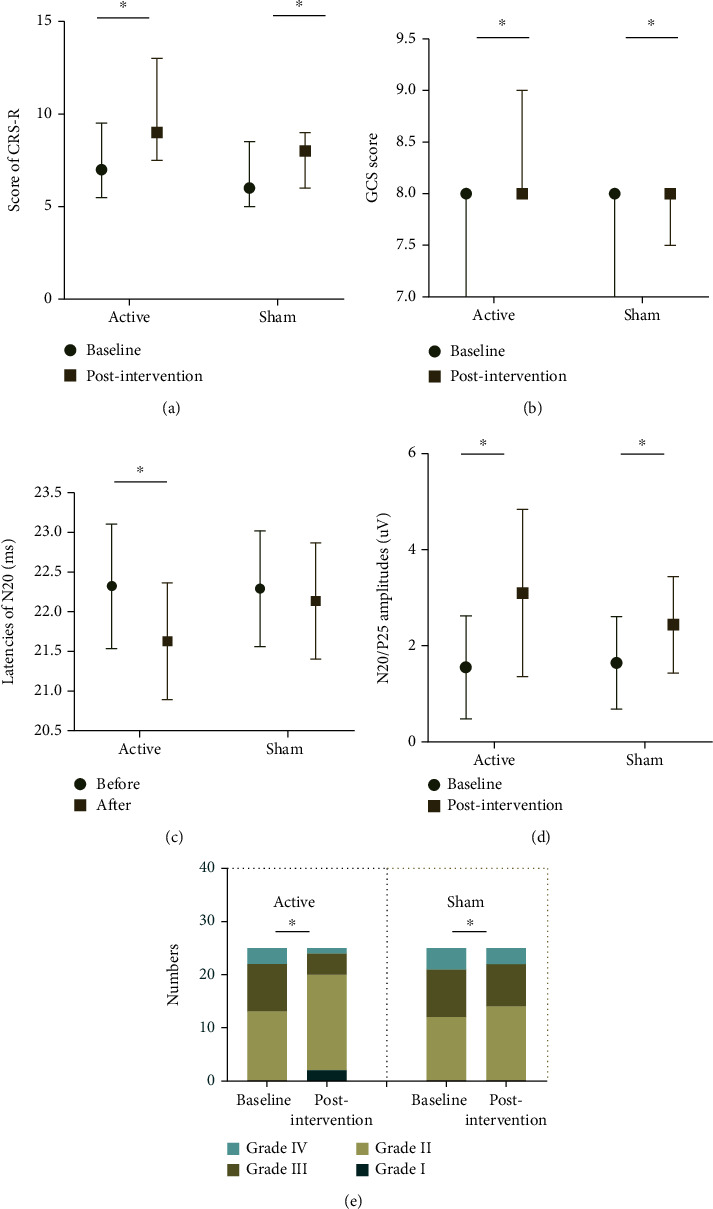
The influence of 30 sessions of active rTMS compared to sham treatment on clinical assessments and electrophysiological evaluation. (a) CRS-R scores poststimulation in both groups were significantly increased compared to that in baseline; (b) GCS scores poststimulation in both groups were significantly increased compared to that in baseline; (c) N20 latencies following active rTMS were significantly shorter compared to that in baseline, while no significant decrease was produced in the sham group compared with baseline; (d) N20-P25 amplitudes in both groups were significantly higher compared to that in baseline; (e) BAEP grades in both groups were significantly improved compared to that in the baseline. ^∗^*p* < 0.05. Abbreviations: CRS-R: Coma Recovery Scale-Revised; GCS: Glasgow Coma Scale.

**Figure 4:**
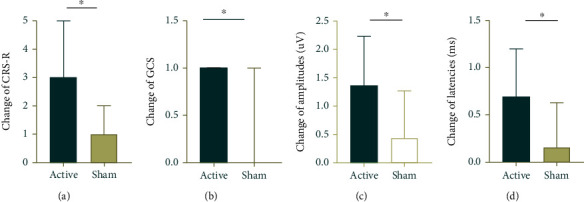
Changes in scores of clinical scales and electrophysiological parameters from baseline to postintervention for both groups. HF-rTMS produced a greater elevation of (a) CRS-R scores and (b) GCS scores in the active group than in the sham group; HF-rTMS produced (c) a substantial increase of N20-P25 amplitudes and (d) a substantial decrease of N20 latencies in the active group compared with the sham group.

The total CRS-R score improved significantly at the end of the 6-week interventions compared to baseline in the active group and the sham group (*p* value < 0.001 for both conditions). The improvements in the total GCS score were also considered with both the real (*p* value < 0.001) and sham stimulation (*p* value = 0.007) (Table 2 and Figure 3). The changes in score in total CRS-R score (*p* value = 0.001) and GCS score (*p* value = 0.014) were significantly higher in the active group than in the sham group postintervention (Table 3 and Figure 4). The scores for components of GCS and CRS-R scale in each group were provided in the Supplementary Materials (available here).

### 3.2. The Effects of rTMS on Electrophysiological Assessment

N20-P25 amplitudes (all *p* value < 0.001) and BAEP grade (*p* value = 0.022 vs. *p* value = 0.013) showed significant improvement in patients who received active rTMS at postintervention in comparison to baseline. Latencies of N20 improved significantly at postintervention compared to baseline in the active group (*p* value < 0.001), but not in the sham group (*p* value = 0.113) (Table 2 and Figure 3). The changes in latencies of N20, N20-P25 amplitudes, and BAEP grade were significantly different between the active and sham stimulation conditions (*p* value = 0.018, *p* value = 0.011, and *p* value = 0.013, respectively). The details are summarized in Table 3 and Figure 4.

### 3.3. The Relationship between Clinical Assessments and Electrophysiological Parameters

**Figure 5:**
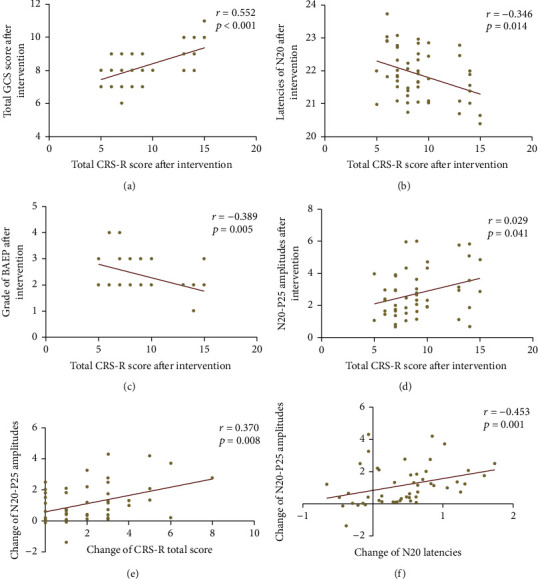
Correlation between the scores of clinical scales and the parameters of electrophysiological assessments after intervention. (a) The total GCS score was significantly related to the total CRS-R score. (b) The N20 latency exhibited a significant negative correlation with the total CRS-R score. (c) The BAEP grade was related to the total CRS-R score. (d) The total CRS-R score was related to the N20-P25 amplitude. The change of N20-P25 amplitude was related to (e) the change of total CRS-R score and (f) N20 latency.

A strong and significant positive correlation was found between the total CRS-R score and the total GCS score postintervention in all patients (*r* = 0.552, *p* value < 0.001). The latency of N20 at postintervention in all patients exhibited a significant negative correlation with the total CRS-R score (*r* = −0.346, *p* value = 0.014). The grade of BAEP after interventions was related to the total CRS-R score (*r* = −0.339, *p* value = 0.016). The N20-P25 amplitude after interventions was related to the total CRS-R score (*r* = 0.0291, *p* value = 0.041). The changes in N20-P25 amplitude before and after interventions were related to the changes in total CRS-R score (*r* = 0.370, *p* value = 0.008) and latency of N20 (*r* = 0.453, *p* value = 0.001). The details are summarized in Figure 5.

## 4. Discussion

As a representative of noninvasive brain stimulation (NIBS) techniques, transcranial magnetic stimulation (TMS) has been viewed as a potential experimental approach to DOC treatment, attracting increasing attention [13, 31]. Despite neurobehavioral gains in some research and clinical settings, there is a paucity of evidence regarding the effects of its application on neural activity [33]. Therefore, the present randomized controlled clinical study was performed using electrophysiological and neurobehavioral assessments to explore clinical neurophysiological evidence in consciousness recovery during therapy according to an HF-rTMS protocol in patients with DOC. The results show that HF-rTMS can produce detectable electrophysiological modifications in DOC patients. There was also improvement in the CRS-R and GCS scores following six weeks of HF-rTMS to the DLPFC. More importantly, the findings of the electrophysiological assessments were, to some extent, compatible with the scores of clinical neurobehavior.

The response to rTMS is mediated by the brain network that is preserved after insult. When neural connectivity is preserved, the thalamocortical system should respond to TMS with a complex activation pattern, involving various cortical areas; on the contrary, after losing connectivity, TMS pulses only produce a simple activation localized to the stimulation site [33]. It is worth noting that the N20 and P25 components in SSEP are the primary cerebral responses to electrical stimulation applied to median nerves [45]. The presence of the bilateral N20 and P25 components at baseline, especially the amplitude from N20 to P25, may be a strong predictor of return to consciousness in DOC patients [25, 26, 46], showing preservation of neural pathway connectivity [31]. Bagnato et al. showed that N20-P25 amplitudes are related to consciousness recovery [26]. In the report by Naro et al., the effect of a single session of rTMS is only shown in DOC patients with bilateral N20 [13]. The residual neural pathway is capable of reacting as an efficient substrate for rTMS [12]. Therefore, the SSEP may make it possible to select patients eligible for rTMS. In this study, the presence of bilateral N20 and P25 was determined by SEP in all patients before enrollment in the study. The presence of bilateral N20 and P25 in these patients could suggest that they may have a greater likelihood of recovery at baseline. In the present study, we also observed the improvements in clinical behavior scales assessed by the CRS-R and GCS in both groups at postintervention when compared to baseline.

A disruption of interregional neural connectivity is associated with a breakdown in consciousness [9]. Neural functional connectivity is an important characteristic to consider when describing consciousness levels [5, 11]. The generation and regulation of consciousness are heavily dependent on specific sensory input through thalamocortical pathways [36], and the connectivity of the pathways can be evaluated by the parameters of SSEP [28, 43]. Keren et al. reported that dynamic changes of N20 in amplitudes and latencies can be related to the changes in consciousness conditions in unaware patients [18]. On the other hand, actions of the ascending reticular activating system (ARAS) which is predominately located in the midbrain and pons also play a significant role in the maintenance of consciousness. The connectivity of the brainstem network could reflect its capability to propagate ARAS signals throughout the cortex [32], which could then be assessed by BAEP [44]. The patterns of five consecutive neurogenic waves in BAEP are closely related to specific neuroanatomical structures in the auditory pathway, including the cochlear nerve, cochlear nucleus, olivary complex, lateral lemniscus, and inferior colliculus [47]. The presence or absence of these waves, their bilateral symmetry for parallel construction, and their characteristics are also often used to evaluate the severity and prognosis of DOC [17, 48]. In our study, SEP and BAEP were used to evaluate the connectivity of neural pathways and the severity of DOC. Along with the gains in clinical neurobehaviors, we also observed improvements in electrophysiological parameters for these patients at postintervention compared to baseline, particularly in the amplitudes of the N20-P25 and BAEP grades. These results indicate significant normalization of functional neural connectivity after stimulation treatment. Interestingly, Pisani et al. [28] showed that the degree of neural functional connectivity is proportionally related to the consciousness level in patients suffering from DOC. In the present study, significant relationships were also observed between higher behavioral performance (CRS-R scores) and better levels of neural pathway connectivity including the latency of N20 and BAEP grade. The obvious positive relationship between the CRS-R score and the amplitude of N20-P25 was also observed.

The induced effects of rTMS depend, in part, on the parameters of stimulation used. As such stimulation at high frequencies (>5 Hz) can induce neural excitation, the frequency commonly utilized in previous studies ranged from 5 to 20 Hz [21, 31, 38]. Moreover, repetitive TMS may induce more significant perturbations in contrast to single TMS, with deep and sustained effects on subcortical regions [49] that can be maintained long after completing rTMS sessions [36]. Given the risk for seizure induction, the effective stimulation frequency in the rTMS design used for this study is repetitive stimulation at 10 Hz with 90% RMT. In addition, Louise-Bender et al. have highlighted the therapeutic effect of 10 Hz rTMS, concluding that in DOC patients, 30 applications may promote clinically significant neurobehavioral recovery [36]. Therefore, 30 sessions of rTMS were performed in the present study, and active rTMS produced a greater elevation of changes in total CRS-R and GCS scores compared to sham stimulation. Notably, no side effects were observed for any of our patients either during or after the entire experiment. The negative results in the improvement of clinical assessment in the study by Naro et al. may be due to the use of only a single session of 10 Hz rTMS in DOC patients [13].

The results from the present study suggest that the underlying mechanisms for behavioral gains could be attributed to the improved connectivity efficiency of a neural pathway. It has been proposed by Pisani et al. that rTMS is capable of modulating the efficient functional connectivity for the neural networks through long-term potentiation like synaptic plasticity mechanisms [28]. Jane et al. observed that the volume of the neural tract of the right prefrontal cortex increased in concert with the provision of comprehensive rehabilitation including rTMS for months by using serial diffusion tensor tractography in a DOC patient in a clinical setting. Several basic studies have also shown that rTMS can remodel dendritic spines by promoting neuronal plasticity related to genes and protein expression [50]. Hence, the improved neural connectivity could be related to the additional recruitment of dendritic (presynaptic or postsynaptic) plasticity by rTMS [28]. The reconstruction of neural connectivity depends not only on local nerve regeneration, but also on effective stimulation of remaining nerve fibers in the damaged area to maximize their use [51]. Our results in this study show that active rTMS significantly decreased the latencies of N20 and elevated the N20-P25 amplitudes compared to the sham stimulation; the latencies of N20 also improved significantly at postintervention compared to the baseline in the active group but not the sham group. Many studies have found that neurophysiological changes after sessions of rTMS in patients with prolonged DOC are related to clinical improvements [33, 36]. In this study, our results are consistent with previous observations that 30 sessions of rTMS altered neural functional connectivity and result in improved behavioral performance and that a positive correlation was observed between the change in CRS-R score and N20-P25 amplitude.

The present sham-controlled study of 50 patients with bilateral N20 receiving real or sham rTMS stimulation for 30 sessions revealed higher behavioral gains (total CRS-R and GCS scores), as well as more significant improvement in the electrophysiological parameters (latencies of N20 and N20-P25 amplitudes and BAEP grades) of patients following real rTMS stimulation compared to those receiving sham stimulation. These findings indicate that preserved neural connectivity may be a key point of consciousness recovery in severe DOC patients. The residual plasticity potentiality can be properly triggered by rTMS to elevate neural connectivity and improve the level of consciousness for DOC patients. Future studies with larger sample sizes and the stratification of patients should be carried out to explore whether rTMS might also induce effects in patients with one or without the N20 component by other quantitative assessment means. In addition, this study also has certain limitations. First, the small sample size was largely due to the difficulty of finding eligible patients for such a long study. Second, the present study was a monocentric study. Third, the study did not investigate how long the rTMS-induced effects could last or the long-term prognosis for patients. The prognosis may related to many factors including family, economy, transfer, length of hospital stays, and subsequent treatment levels. Finally, the patient population was heterogeneous, representing patients with different kinds of lesions and diagnoses.

## 5. Conclusion

In conclusion, rTMS could be a promising treatment strategy for DOC. The 10 Hz rTMS over the right DLPFC can effectively modulate neural functional connectivity and increase behavioral performance in DOC patients with the presence of bilateral N20 in a short term. Our preliminary results indicate that Eps might be useful for the assessment of the effects of rTMS, and an elevation in the connectivity of neural pathways may be one important potential mechanism of rTMS on DOC. However, this is a preliminary study in DOC patients with bilateral N20. Larger studies are needed to confirm the long-term effects and determine the safety in other DOC populations.

## Figures and Tables

**Table 1:** Baseline characteristics of the study population.

Parameters	Active group (*n* = 25)	Sham group (*n* = 25)	*p* value	95% CI
Age (year)	50.520 ± 13.857	52.60 ± 14.396	0.772^a^	-10.12, 5.96
Duration of disease (month)	3 (1-3)	2 (1-3)	0.803^b^	—
Sex (number)				
Male	18	16	0.762	
Female	7	9		
Etiology (number)				
TBI	12	9	0.353	—
Stroke	10	9		
Anoxia	3	7		
Baseline clinical assessment				
CRS-R total score	7.160 ± 2.285	6.880 ± 2.279	0.630^b^	—
GCS total score	8 (7-8)	8 (7-8)	0.763^b^	—
Baseline electrophysiology examination				
Latencies of N20 component (ms)	22.321 ± 0.785	22.290 ± 0.729	0.923^b^	—
N20-P25 amplitudes (*μ*V)	1.556 ± 1.070	1.643 ± 0.965	0.761^a^	-0.668, 0.492
BAEP grade (number)				
Grade-I	0	0	0.717^b^	—
Grade-II	13	12		
Grade-III	9	9		
Grade-IV	3	4		

Data are presented as mean ± standard deviation or median (interquartile range). Abbreviations: TBI: traumatic brain injury: CRS-R, Coma Recovery Scale-Revised; GCS: Glasgow Coma Scale; BAEP: brainstem auditory-evoked potential; CI: confidence intervals. Significance level at *p* value < 0.05; *p* value refers to the results of the ^a^independent samples *t*-test, the ^b^Mann–Whitney *U* test, and the ^c^Chi-squared test.

**Table 2:** Comparison measured parameters at baseline and postintervention in the active and sham groups.

Variable	Baseline	Postintervention	*p* value	95% CI
CRS-R total score				
Active group	7.160 ± 2.285	9 (7.5-13)	<0.001^b^	—
Sham group	6.880 ± 2.279	8 (6 -9)	<0.001^b^	—
GCS total score				
Active group	8 (7-8)	8 (8-9)	<0.001^b^	—
Sham group	8 (7-8)	8 (7.5-8)	0.007^b^	—
Latencies of N20 component (ms)				
Active group	22.321 ± 0.785	21.627 ± 0.733	<0.001^a^	0.490, 0.900
Sham group	22.290 ± 0.729	22.134 ± 0.733	0.113^a^	-0.040, 0.350
N20-P25 amplitudes (*μ*V)				
Active group	1.556 ± 1.070	3.099 ± 1.744	<0.001^a^	-2.012, -1.076
Sham group	1.643 ± 0.965	2.436 ± 1.007	<0.001^a^	-1.163, -0.422
BAEP grade (number)				
Active group (grade-I/II/III/IV)	0/13/9/3	2/18/4/1	0.022^b^	—
Sham group (grade-I/II/III/IV)	0/12/9/4	0/14/8/3	0.013^b^	—

Data are presented as mean ± standard deviation or median (interquartile range). Abbreviations: CRS-R: Coma Recovery Scale-Revised; GCS: Glasgow Coma Scale; BAEP: brainstem auditory-evoked potential; CI: confidence intervals. Significance level at *p* value < 0.05; *p* value refers to the results of ^a^two-tailed unpaired Student's *t*-test and the ^b^paired Wilcoxon rank-sum test.

**Table 3:** Changes in measured parameters from baseline to postintervention for patients in the active and sham groups.

Variable	Change (postintervention—Baseline)	*p* value
CRS-R total score		
Active group	3.0 ± 2.082	0.001^a^
Sham group	1.0 (0-2)	
GCS total score		
Active group	1 (0-1.5)	0.014^a^
Sham group	0 (0-1)	
Latencies of N20 component (ms)		
Active group	0.694 ± 0.504	0.018^b^
Sham group	0.155 ± 0.472	
N20-P25 amplitudes (*μ*V)		
Active group	1.360 (0.510-2.235)	0.011^a^
Sham group	0.440 (0.115-1.265)	
Change in BAEP grade (number)		
Active group (no improvement/improvement one grade)	14/11	0.013^a^
Sham group (no improvement/improvement one grade)	22/3	

Data are presented as mean ± standard deviation or median (interquartile range). Abbreviations: CRS-R: Coma Recovery Scale-Revised; GCS: Glasgow Coma Scale; BAEP: brainstem auditory-evoked potential. Significance level at *p* value < 0.05; *p* value refers to the results of the ^a^Mann–Whitney *U* test and the ^b^independent samples *t*-test.

## Data Availability

The data that support the findings of this study are available from the corresponding author upon reasonable request.

## Abbreviations

DOC: Disorders of consciousness
Eps: Evoked potentials
HF-rTMS: High-frequency repetitive transcranial magnetic stimulation
SEP: Somatosensory-evoked potential
BAEP: Brainstem auditory-evoked potential
CRS-R: Coma Recovery Scale-Revised
CGI-I: Clinical Global Impression Improvement
GCS: Glasgow Coma Scale
DLPFC: Dorsolateral prefrontal cortex
VS: Vegetative state
MCS: Minimally conscious state
MEP: Motor-evoked potentials
TMS: Transcranial magnetic stimulation
FDI: First dorsal interosseous
RMT: Resting motor threshold
NIBS: Noninvasive brain stimulation
ARAS: Ascending reticular activating system.

## Supplementary Materials

Supplementary Materials Figure S1 The sample plots of somatosensory-evoked potential (SEP) for one subject in the TMS group before and after intervention. Figure S2 The sample plots of SEP for one subject in the sham group before and after intervention. Figure S3 The sample plots of brainstem auditory-evoked potentials (BAEP) for one subject in the TMS group before and after intervention. Figure S4 The sample plots of BAEP for one subject in the sham group before and after intervention. Table S1 Parameters of somatosensory-evoked potential (SEP) and brainstem auditory-evoked potential (BAEP) for the sample plots. Table S2 Demographic details of the patients
