# Supplementary material for: Influence of High-Frequency Repetitive Transcranial Magnetic Stimulation on Neurobehavioral and Electrophysiology in Patients with Disorders of Consciousness
Source: Neural Plast. 2022 Nov 16;2022:7195699. doi: 10.1155/2022/7195699 (PMC9699789; doi:10.1155/2022/7195699)
Supplement: Supplementary Materials — Figure S1 The sample plots of somatosensory-evoked potential (SEP) for one subject in the TMS group before and after intervention. Figure S2 The sample plots of SEP for one subject in the sham group before and after intervention. Figure S3 The sample plots of brainstem auditory-evoked potentials (BAEP) for one subject in the TMS group before and after intervention. Figure S4 The sample plots of BAEP for one subject in the sham group before and after intervention. Table S1 Parameters of somatosensory-evoked potential (SEP) and brainstem auditory-evoked potential (BAEP) for the sample plots. Table S2 Demographic details of the patients [file 7195699.f1.pdf]

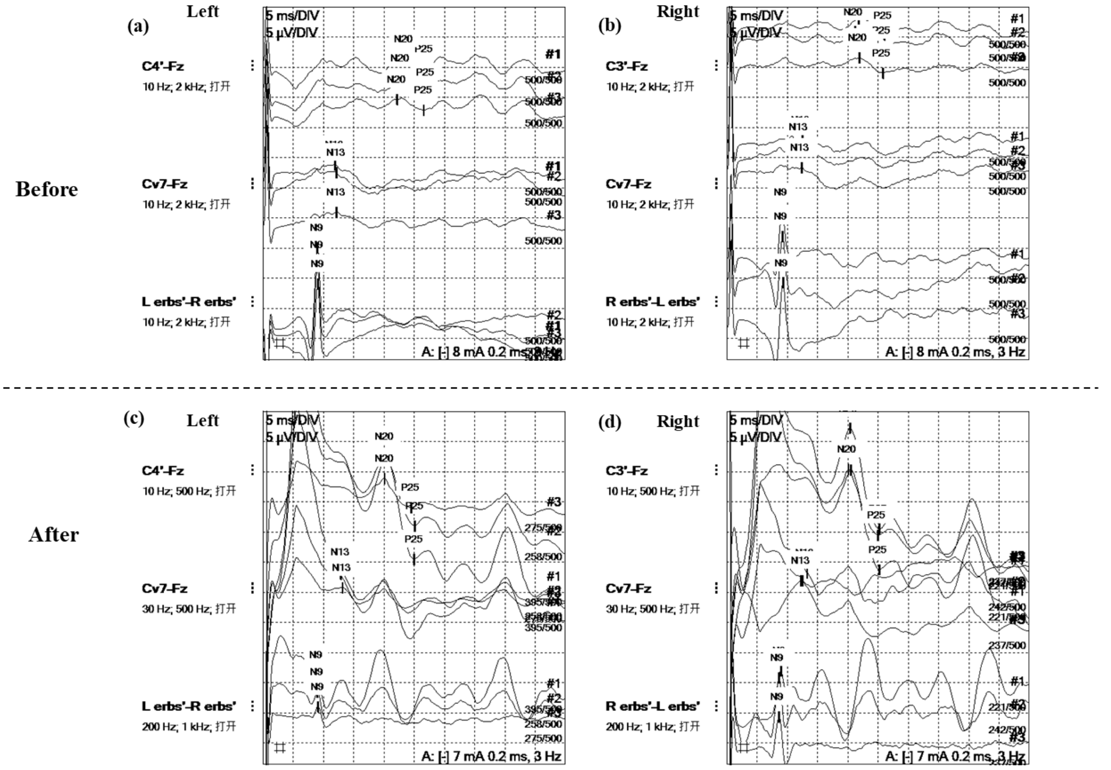

Fig. S1 Example the somatosensory evoked potential (SEP) for one subject in the TMS group. Patients had shorter latency of N20 and higher wave amplitude of N20-P25 after treatment (c and d) compared to pre-treatment (a and b).

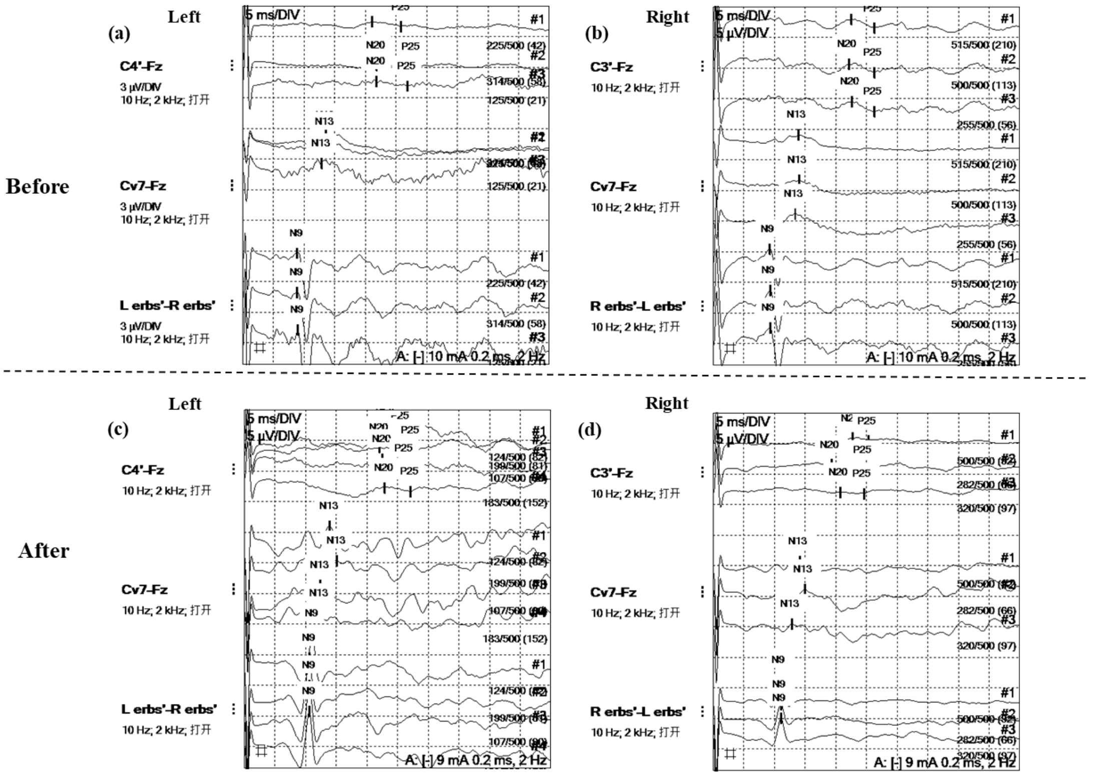

Fig. S2 Example the somatosensory evoked potential (SEP) for one subject in the sham group. No obvious changes

were observed in latency of N20 and wave amplitude of N20-P25 for this patient before (a and b) and treatment after (c and d).

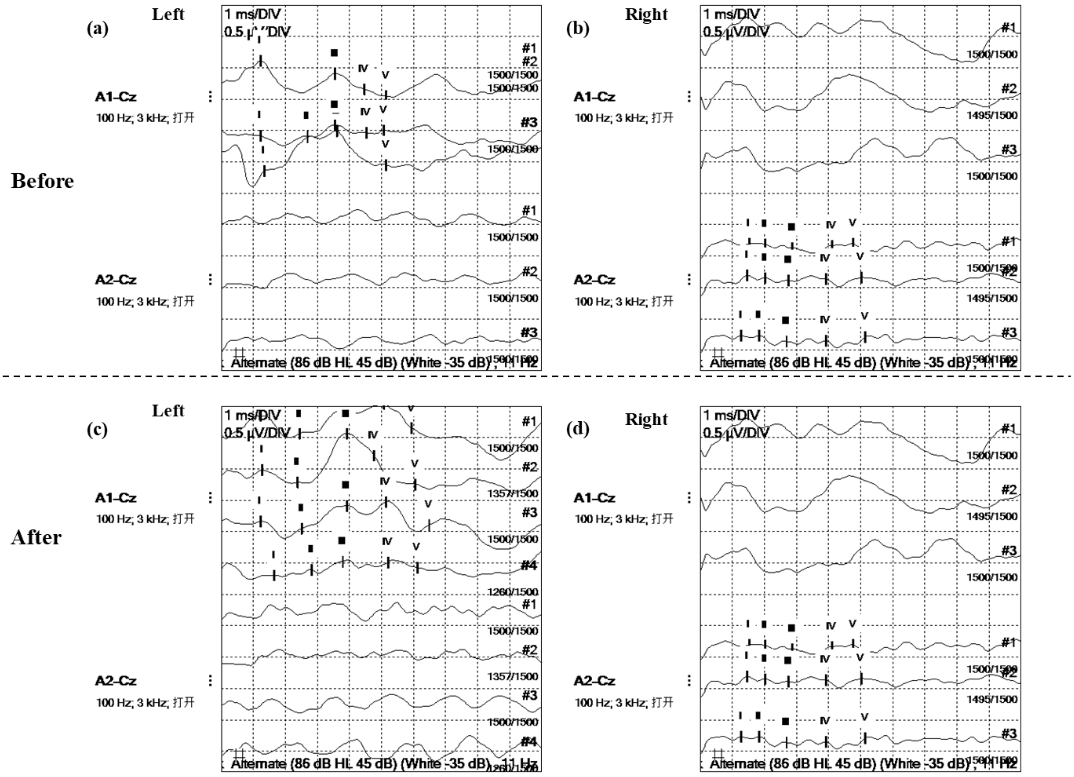

Fig. S3 Example the brain stem auditory evoked potentials (BAEP) for one subject in the TMS group.

The BAEP responses (a and b) were moderate abnormality with poor waveform differentiation before intervention. The waves I to V were mild abnormality with moderate waveform differentiation after intervention (c and d).

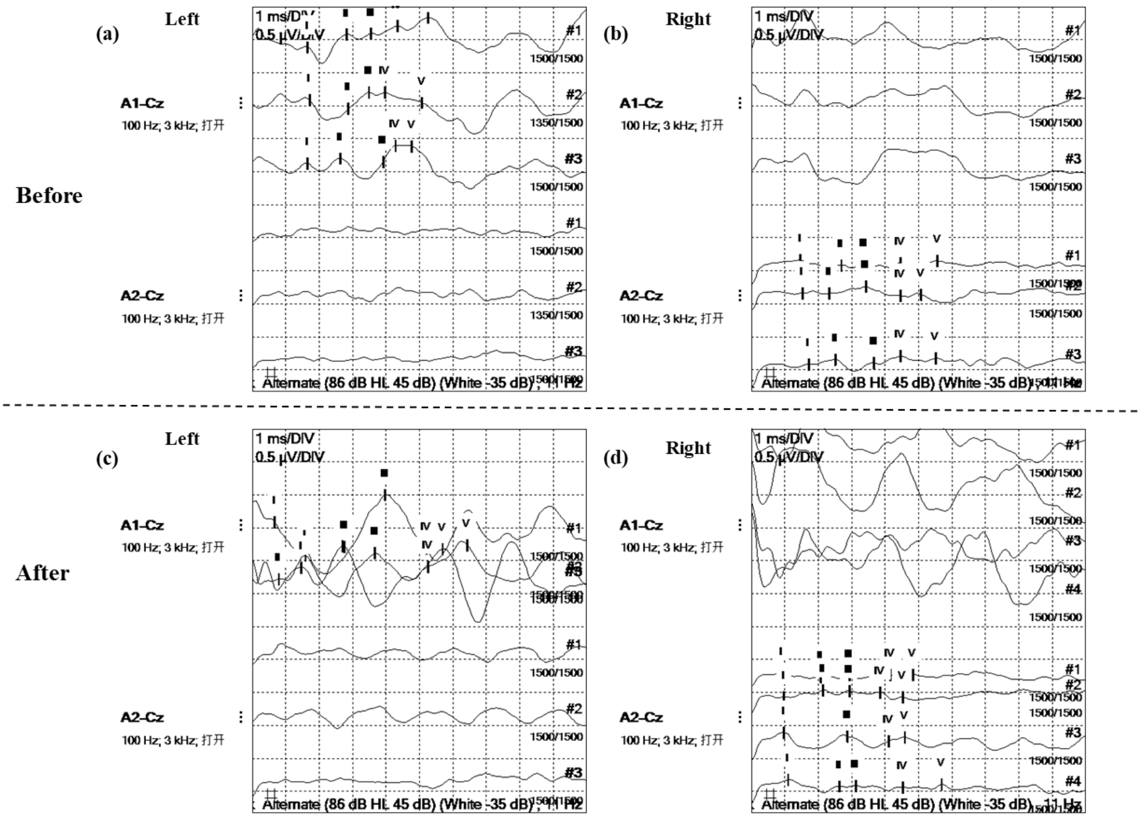

Fig. S4 Example the brain stem auditory evoked potentials (BAEP) for one subject in the sham group. No obvious improvement was observed in waveform differentiation before (a and b) and after intervention (c and d).

Table S1. Parameters of somatosensory evoked potential (SEP) and brain stem auditory evoked potential (BAEP) for the above figures

		SEP				BAEP	
		Latencies of N20 component (ms)		N20-P25 amplitudes (uV)		Grade	
		Left	Right	Left	Right	Left	Right
Real	Before	22.74	21.67	2.53	1.69	III	III
	After	20.22	20.34	10.88	4.87	III	II
Sham	Before	21.59	22.50	1.63	0.50	III	III
	After	22.52	20.97	0.63	0.23	III	III

Table S2. Demographic details of the patients

Group	Patient	Age	Duration	Etiology	CRS-R														GCS							
					Pre-intervention							Post-intervention							Pre-intervention				Post-intervention			
					A	V	M	OM	C	Ar	Total	A	V	M	OM	C	Ar	Total	E	Verbal response	Best motor response	Total	E	Verbal response	Best motor response	Total
TMS group	1	45	3	Stroke	2	1	2	1	0	1	7	2	4	4	2	1	2	15	3	1	3	7	4	1	5	10
	2	48	1	Stroke	1	2	2	1	0	2	8	3	3	3	2	1	2	14	4	1	3	8	4	1	4	9
	3	55	3	TBI	1	2	2	1	0	2	8	2	3	2	2	1	3	13	4	1	3	8	4	1	3	8
	4	56	2	Stroke	2	2	2	1	0	1	8	2	3	3	2	1	3	14	3	1	4	8	4	1	4	9
	5	31	4	Anoxia	1	0	1	0	0	2	4	1	2	2	1	0	2	8	4	1	2	7	4	1	3	8
	6	47	1	TBI	0	0	2	1	0	2	5	1	1	2	1	1	2	8	4	1	2	7	4	1	3	8
	7	50	1	TBI	0	2	2	0	0	2	6	1	2	2	1	1	2	9	4	1	3	8	4	1	3	8
	8	52	6	Stroke	1	2	2	0	0	2	7	1	2	2	1	0	2	8	4	1	3	8	4	1	3	8
	9	70	3	Stroke	1	0	2	0	0	2	5	1	1	2	1	0	2	7	4	1	3	7	4	2	3	9
	10	28	3	TBI	2	2	2	1	0	3	10	3	3	3	2	1	3	15	4	1	3	8	4	3	4	11
	11	34	1	TBI	2	3	2	1	1	3	12	2	3	2	2	1	3	13	4	1	3	8	4	2	3	9
	12	29	1	Stroke	1	2	2	1	1	2	9	2	2	3	2	2	3	14	4	1	3	8	4	2	4	10
	13	56	1	Stroke	1	0	3	0	0	0	4	1	1	3	1	1	2	9	1	1	4	6	4	1	4	9
	14	27	5	TBI	2	2	2	2	0	2	10	2	3	3	2	1	2	13	4	1	3	8	4	2	4	10
	15	57	2	Stroke	0	0	1	1	0	2	4	1	1	2	1	0	2	7	4	1	2	7	4	1	3	8
	16	54	3	TBI	1	2	1	0	0	2	6	1	2	2	1	0	3	9	4	1	2	7	4	1	3	8
	17	60	3	Stroke	2	2	2	1	0	3	10	3	3	2	2	1	2	13	4	1	3	8	4	1	3	8
	18	68	2	TBI	1	0	2	1	0	2	6	1	1	2	1	0	2	7	4	1	3	8	4	1	3	8
	19	68	1	TBI	1	0	2	2	0	2	7	1	1	2	2	0	2	8	4	1	3	8	4	2	3	9

	20	65	1	TBI	2	2	2	2	0	2	10	2	2	2	2	0	2	10	4	1	3	8	4	1	3	8
	21	33	2	Anoxia	0	0	1	1	0	2	4	1	1	1	1	1	2	7	4	1	2	7	4	1	2	7
	22	45	3	Anoxia	1	2	1	0	0	2	6	1	1	1	0	0	2	7	4	1	2	7	4	1	3	8
	23	50	3	TBI	2	3	1	1	0	3	10	2	3	1	1	0	3	10	4	1	3	8	4	1	3	8
	24	63	4	TBI	1	0	2	1	0	2	6	1	1	2	1	0	2	7	4	1	3	8	4	1	3	8
	25	72	4	Stroke	1	0	2	2	0	2	7	1	1	2	1	0	2	9	4	1	3	8	4	2	3	9
Sham group	1	33	2	TBI	2	2	3	1	0	2	10	2	3	3	2	1	2	13	4	1	4	9	4	1	4	9
	2	35	6	Anoxia	1	1	1	0	0	2	5	2	1	1	1	0	2	7	4	1	2	7	4	1	2	7
	3	73	6	Stroke	1	1	2	1	0	1	6	2	1	2	1	0	1	7	4	1	3	8	4	1	3	8
	4	79	1	Anoxia	0	0	2	1	0	1	4	1	1	2	2	0	1	7	1	1	3	5	2	1	3	6
	5	50	1	Stroke	1	1	2	1	0	1	6	1	1	2	1	0	1	6	4	1	3	8	3	1	4	8
	6	58	4	TBI	1	1	1	1	0	1	5	1	1	2	1	0	1	6	3	1	2	6	3	1	3	7
	7	66	1	Stroke	1	1	2	1	0	1	7	2	2	2	1	0	1	9	3	1	3	7	3	1	3	7
	8	55	1	TBI	1	2	2	1	0	2	8	2	2	2	1	0	2	9	4	1	3	8	4	1	3	8
	9	41	6	TBI	1	2	2	1	0	2	8	2	2	2	1	0	2	9	4	1	3	8	4	1	3	8
	10	52	3	Stroke	2	2	2	1	0	1	8	2	2	2	1	0	1	8	3	1	4	8	4	1	4	9
	11	44	3	Anoxia	1	0	1	0	0	2	4	1	1	1	0	0	2	5	4	1	2	7	4	1	3	8
	12	67	1	TBI	0	0	2	1	0	2	5	2	1	2	1	0	2	8	4	1	2	7	4	1	2	7
	13	66	1	TBI	0	2	2	0	0	2	6	2	2	2	1	0	2	9	4	1	3	8	4	1	3	8
	14	70	1	Stroke	1	2	2	0	0	2	7	1	2	2	1	0	2	8	4	1	3	8	4	1	3	8
	15	45	5	Stroke	1	0	2	0	0	1	5	1	0	2	0	0	2	5	3	1	3	7	4	1	2	7
	16	51	2	TBI	2	2	2	1	0	3	10	2	2	2	1	0	3	10	4	1	3	8	4	1	3	8
	17	47	3	TBI	2	3	2	1	1	3	12	3	3	2	2	1	3	14	4	1	3	8	4	1	3	8
	18	58	3	Stroke	1	2	2	1	1	2	9	1	2	2	1	1	2	9	4	1	3	8	4	2	3	9
	19	64	2	Stroke	1	0	3	0	0	0	4	1	1	3	1	1	1	6	1	1	4	6	2	1	4	9

	20	52	1	TBI	2	2	2	2	0	2	10	2	2	2	2	0	2	10	4	1	3	8	3	1	4	8
	21	42	1	Anoxia	0	0	1	1	0	2	4	1	1	1	1	0	2	6	4	1	2	7	4	1	3	8
	22	40	2	Anoxia	1	2	1	0	0	2	6	1	2	1	0	0	2	6	4	1	2	7	4	2	2	8
	23	34	3	Stroke	2	3	1	1	0	3	10	2	3	1	1	0	3	10	4	1	3	8	4	2	2	8
	24	70	3	Anoxia	1	0	2	1	0	2	6	1	1	2	1	0	2	7	4	1	3	8	4	1	3	8
	25	23	2	Anoxia	1	0	2	2	0	2	7	2	1	2	2	0	2	9	4	1	3	8	4	1	3	8

Abbreviations: TBI: traumatic brain injury, CRS-R, Coma Recovery Scale-Revised; A, auditory; Ar, arousal; C, communication; M, motor; OM, oro-motor; V, visual; GCS: Glasgow Coma Scale; E, eye-opening.
